# Editorial: Oncolytic Viruses—Genetically Engineering the Future of Cancer Therapy

**DOI:** 10.3389/fonc.2017.00271

**Published:** 2017-11-13

**Authors:** Benjamin Gesundheit, Joshua P. Rosenzweig

**Affiliations:** ^1^Rapo Yerapeh Ltd., Jerusalem, Israel

**Keywords:** oncolytic viruses, oncolytic virotherapy, gene therapy, immune checkpoint blockade, antitumor immunity, cancer

**Editorial on the Research Topic**

**Oncolytic Viruses—Genetically Engineering the Future of Cancer Therapy**

Since the 1960s, oncolytic viruses (OVs) have been a target of research as a therapeutic modality for cancer. The mechanism of these viruses involves both direct tumor cell lysis and the induction of immunogenic cell death. Clinical trials have explored a wide variety of viruses including naturally occurring viruses and genetically engineered viruses. Indications have spanned the gamut from hepatocellular carcinoma to soft tissue sarcoma to glioblastoma multiforme to multiple myeloma. Recently, the U.S. Food and Drug Administration announced the first FDA-approved OV therapy, for the treatment of melanoma lesions in the skin and lymph nodes. OVs have been used as single agent therapy or in combination with conventional cancer therapies. Current challenges including both scientific and regulatory do not diminish the significant potential for the future of this modality. Questions about the advantages of one virus over another, the synergistic potential of multiple viruses used in combination, dosage, and optimal route of administration remain unresolved and demand further research. Furthermore, the possibility that a particular OV might be more suitable for a specific cancer type than other OVs depending on the mechanism of the virus and the nature of the cancer raises additional research challenges. The precise role of adjuvant therapies such as dendritic cells in combination with OVs is yet another unresolved area in this innovative field.

While some of the research in this volume focused on specific viruses, others have confined their investigations to specific cancers, the role of the immune system in oncolytic virotherapy, or various strategies in developing recombinant viruses. Ocathail et al. looked at what might be the most familiar and widely understood OV, namely, adenovirus; however, they did not look at the virus in isolation. Rather, they investigated the interaction between the virus and radiation and found that the virus can actually sensitize the tumor to radiation therapy. Yin et al. chose to concentrate on another well-known specific OV, namely, the herpes simplex virus (HSV). They described the particular characteristics that enable HSV to evade T cells and highlighted various strategies in modifying the virus to increase its efficacy along with approaches to combinatorial therapy. Similarly, Eissa et al. also directed their research toward HSV. Their study focused on HF-10, a HSV, which has shown the ability to reduce tumor growth and prolong survival rates. They surveyed the various preclinical and clinical trials with HF-10 in monotherapy and combination therapy. They found that HF-10 has high tumor selectivity and a potent effect against tumors. Shifting to other specific viruses, Kleinlutzum et al. narrowed their investigation to comparing a recombinant measles virus MV-CD133 to a recombinant vesicular stomatitis virus, VSV-CD133. They found that VSV-CD133 infected a much wider area of the tumor than CD133 over the same amount of time. In addition, Angelova et al. focused on a specific virus, namely, the H1 Parvovirus. They reviewed the use of H1 Parvovirus in pancreatic carcinoma and in glioblastoma. They then surveyed the preclinical use of the virus in hematological malignancies specifically. They found that H-1PV can infect and kill cancer cells efficiently in these malignancies. Haddad looked specifically at the vaccinia virus (VACV). She reviewed preclinical studies with genetically engineered VACV strains, analyzing the advantages of this virus as an OV such as its large genomic capacity. Various strategies employed in the newer generations of the engineered viruses were discussed including transgene delivery for treatment, imaging, and combination therapy.

In terms of research on specific cancers Pease and Kratzke honed in on mesothelioma. Since mesothelioma tends to grow locally and in a location that allows for direct injection of the virus into the tumor, we would expect it to be an ideal candidate for oncolytic virotherapy. They summarized the preclinical studies using various viruses for mesothelioma including: adenovirus, HSV-1, vaccinia, measles, and others. Overall, they see much potential for the future for treating mesothelioma with combination approaches including OVs.

The role of the immune system in oncolytic virotherapy is the subject of intense research. Generally, it is assumed that viruses trigger the immune system and that the immune system attacks the tumor cells. Surprisingly, Filley and Dey explain why that is an oversimplification. In fact, the role of the immune system is sometimes actually the opposite. More specifically, there are immunological barriers to oncolytic virotherapy including: neutralizing antibodies, complement proteins, and type I interferon signaling, among others. The vector and timing of the viral infection along with the specific malignancy involved can all play a role in whether the immune system serves as an ally or not in the fight against cancer. Guo et al. also reviewed the nature of the relationship between the immune system and OVs. They highlighted various hurdles preventing OVs from broader use. These included the following: limited range of OVs, premature clearing of viruses by the immune system, and toxicities. Similarly, Holay et al. looked at studies of viruses that have incorporated specific tumor antigens to improve the response of the immune system to the tumors. They suggested that improvements in sequencing, computational techniques, and peptide isolation have enabled better tumor antigen discovery. Jhawar et al. looked at both naturally occurring and engineered viruses and their immune and non-immune pathways. More specifically, they summarized approaches involving improved antigen presentation, heat shock protein, and serotype switching. Meyers et al. focused on three main strategies for developing recombinant viruses. They included: improving host immune response by inserting transgenes such as granulocyte-macrophage colony-stimulating factor, combining OVs with drugs that modulate the immune system such as immune checkpoint inhibitors, and the prime boost strategy. In the prime boost approach, tumor-specific antigens can be built into the one viral platform to prime the immune system before being exposed to a second viral platform carrying the same antigens that upregulate the antitumor immune response. Shifting gears, another extremely innovative strategy in developing recombinant viruses, Bofill et al. investigated the insertion of miRNA response elements recognizing miRNAs expressed in specific tissues, but downregulated in tumors, into viral genes. They explained the complex nature of the interaction between viruses and host cells and how it can be maximized using miRNA. Howells et al. reviewed both gene insertion and gene deletion strategies for generating recombinant OVs. They also reflected on a third strategy involving the control of gene promoters both in tumor cells and in the viral genes.

Irwin et al. analyzed various pathways in the production of deoxynucleotide triphosphate (dNTP). The production of dNTP is often dysregulated in cancer cells. This difference between cancer cells and normal cells can be leveraged to selectively target the cancer cells. They found that the supply of dNTP can affect viral replication and the immune response. Further studies are necessary to explore how viruses can be engineered to capitalize on these findings to improve therapy.

Finally, with the recent success of clinical trials for oncolytic virotherapy, the need to improve methods for monitoring this treatment and to better understand the mechanism of action is great. Ansel et al. reviewed four primary strategies for monitoring oncolytic virotherapy *via* gene expression and highlighted advantages and disadvantages of each one. They concluded that combined gene expression studies looking at both the tumor expression and the viral expression could potentially provide much more information about the efficacy of the treatment modality and its pathway.

## Author Contributions

BG contributed substantially to the conception of the work and revised it critically. JR contributed substantially to the conception, design, and analysis of the work and also drafted the work.

## Conflict of Interest Statement

BG and JR are employees of Rapo Yerapeh Ltd.

